# Data-Driven Based Prediction and Optimization of Balling Levels in Laser Powder Bed Fusion Additive Manufacturing

**DOI:** 10.3390/ma18091949

**Published:** 2025-04-25

**Authors:** He Qiu, Guo-Zhang Jiang, Xin Lin

**Affiliations:** 1 Precision Manufacturing Institute, Wuhan University of Science and Technology, Wuhan 430081, China; wustqiuhe@gmail.com (H.Q.); whjgz@wust.edu.cn (G.-Z.J.); 2 Key Laboratory of Metallurgical Equipment and Control Technology, Ministry of Education, Wuhan University of Science and Technology, Wuhan 430081, China; 3 Hubei Key Laboratory of Mechanical Transmission and Manufacturing Engineering, Wuhan University of Science and Technology, Wuhan 430081, China

**Keywords:** laser powder bed fusion, data-driven model, process parameters optimization, balling levels

## Abstract

Laser powder bed fusion has been demonstrated as a promising additive manufacturing technology due to its unique advantages, such as weight reduction, the ability to produce arbitrarily complex geometries and single-step manufacturing. However, the production quality may deteriorate due to the poor surface quality of deposited layers caused by the occurrence of the balling phenomenon, which hampers its widespread application. In this work, a data-driven framework is proposed to optimize the process parameters of laser powder bed fusion to achieve satisfactory balling levels. The effects of key process parameters on balling levels are also investigated. Specifically, an image segmentation-based method is introduced to quantitatively evaluate the balling levels on the interlayer surfaces of as-built specimens under various process parameter combinations. Considering the limited amount of experimental data, different machine learning models, including polynomial regression, support vector regression, and backpropagation neural networks, are developed to predict the balling levels within a predefined process parameter space. The predicted values from the best-performing model are then used as fitness values of individuals in an improved genetic algorithm to search for globally optimal process parameters. The final validation experiments confirm that the as-built parts fabricated using the optimized process parameters exhibit minimal balling levels, demonstrating the effectiveness and feasibility of the proposed framework for balling level prediction and optimization. This study provides valuable insights and practical guidance for enhancing the quality of specimens produced in the laser powder bed fusion process.

## 1. Introduction

Laser Powder Bed Fusion (LPBF), as one of the most promising additive manufacturing technologies, enables the direct fabrication of 3D metal components from metal powders without being constrained by the geometric shape and structure of the parts [1,2]. The fundamental principle of this process involves using a scanning laser beam to selectively melt thin layers of metal powder based on sliced data derived from the 3D CAD model of the part. Following a “bottom-up” approach, the process achieves rapid fabrication of metal components through layer-by-layer accumulation. Compared with traditional subtractive and mass-conserving manufacturing techniques, LPBF offers advantages such as lower time cost, the ability to fabricate complex thin-walled components, and no need for post-processing, and it has been widely applied in industries such as automotive, electronics, robotics, and aerospace [3,4,5].

However, LPBF is a complex physical and chemical metallurgical process, and its forming process involves various forms of heat, force, and momentum transfer [6]. During the laser scanning process, metal powders undergo rapid melting and solidification, with large thermal gradients causing a series of defects that hinder the successful fabrication of high-quality metal components with desirable microstructures and properties. These defects mainly include porosity, deformation, delamination, etc., among which the balling phenomenon is particularly prominent. Generally, the balling phenomenon is macroscopically manifest as the aggregation of metal pellets within a single layer, mainly caused by insufficient melting of the metal powder and spatter formation [7]. The occurrence of the balling phenomenon will significantly increase the surface roughness of the parts and disrupt the continuity of the forming process [8]. In addition, it can also induce the generation of internal defects such as lack of fusion, porosity, cracks, and delamination [9] during the continuous forming process. This is primarily because the balling particles severely impede the uniform deposition of fresh powder onto previously sintered layers. Similar to traditionally manufactured parts, the microstructures caused by the balling phenomenon negatively impact the mechanical properties of LPBF-fabricated components, thereby limiting their application in high-strength and fatigue-resistant scenarios [10,11,12,13].

To eliminate or mitigate the balling phenomenon in metal systems, scholars gradually pay attention to the balling defect itself and try to improve the quality of LPBF-formed parts by understanding the formation mechanism of the balling phenomenon and its dependence on process parameters. Regarding the formation mechanism, Childs [14] and Yadroitsev [15] et al. attributed the formation of balling defects in selective laser melting (SLM) to the Plateau-Rayleigh capillary instability of the molten liquid based on theoretical analysis of experimental results. Using the finite volume method (FVM) to simulate melt pool dynamics in SLM, Dai and Gu et al. [16] identified thermocapillary forces and recoil pressure induced by vaporization as the primary driving forces behind spattering-induced balling. Similarly, Zöller et al. [17] applied the Smoothed Particle Hydrodynamics (SPH) method to numerically study the balling defects in the formation of single-track during the laser powder bed fusion of Inconel 718. Their findings revealed that the balling phenomenon was predominantly driven by Marangoni forces and capillary forces. Besides, the emergence of the balling phenomenon can also be attributed to the competitive processes between melt solidification and diffusion in metal systems. Zhou et al. [18] investigated the balling phenomenon of tungsten (W) parts processed by SLM and concluded that this competition between melt diffusion and solidification is governed by the inherent properties of tungsten, as well as the laser processing parameters employed. Due to tungsten’s high thermal conductivity, high liquid viscosity, and significant surface tension, the molten droplets solidify before fully diffusing, thereby maintaining their spherical geometry. In contrast to the competitive mechanisms observed in metal systems, the balling phenomenon during the preparation of alumina ceramics is primarily governed by the diffusion process. Qiu et al. [7] found that the balling phenomenon still occurs even when alumina ceramic powders are fully melted without spattering, indicating that the diffusion process is the dominant factor behind such balling behavior.

As for the dependence of balling defects on process parameters, most current researches focus on controlling the balling particles from the perspective of SLM processing, such as adjusting process parameters [9,19]. It is believed that the adjustment of process parameters will affect the flow stability and surface tension, which, in turn, affects the phenomenon of balling. For instance, Jia et al. [20] investigated the surface morphology and density of Inconel 718 components fabricated under different process parameters. They alleviated the balling phenomenon and achieved improved surface morphology and higher density by reducing the scan speed or increasing the laser power to enhance the laser energy density. Li et al. [21] effectively mitigated the balling phenomenon during the forming process by lowering the oxygen content in the forming chamber to 0.1%. Furthermore, they found that when the scanning trajectory was continuous, the impact of hatch space on the balling phenomenon was negligible. Gu et al. [22] studied the balling phenomenon and its control method for 316L stainless steel powders in the process of direct metal laser sintering (DMLS). They discovered that increasing the volumetric energy density (VED)—by raising the laser power, decreasing the scan speed, or reducing the powder layer thickness—could reduce the tendency of balling. Additionally, they observed that incorporating a small amount of deoxidizing agents into the powder resulted in a smooth, balling-free sintered surface. Yu et al. [23] simulated the single-track forming process of AlSi10Mg and found that appropriately increasing the laser power allowed for the complete melting of the metal powder, thereby improving wettability and surface quality. However, they also noted that excessive laser power induced porosity and exacerbated balling effects, leading to rough surfaces. This is mainly because insufficient laser power fails to fully melt the powder, while excessive power destabilizes the melt pool and leads to self-balling. The above-mentioned studies demonstrate that the severity of the balling phenomenon is closely related to process parameters. Properly controlling these parameters can effectively prevent the occurrence of balling, ultimately enabling the production of high-quality components.

As mentioned in the previous section, to identify optimal process parameters for achieving high-quality fabrication of components, one method is to establish a physics-based numerical model or conduct trial-and-error experiments to analyze the effects of process parameters. The former requires a deep understanding of the physical principles underlying the LPBF process, which can be challenging, especially when only partial information about the process is available. The latter, on the other hand, involves extensive experimentation, which is often time-consuming and labor-intensive. Another approach is to seek an alternative numerical prediction method based on a small amount of data. To this end, numerous researchers in the field of additive manufacturing have focused on using statistical methods or machine learning techniques to quantify the relationship between process parameters and the response variables related to forming quality, such as density, surface roughness, dimensional accuracy, and fatigue life, and using different evolutionary computation methods to optimize the process parameters to achieve high-quality fabrication of specimens. For example, Costa et al. [24] aimed to maximize the density of SLMed Ti6Al4V parts and constructed a predictive model based on the response surface methodology (RSM) and artificial neural network (ANN) to characterize the complex relationship between process parameters and density. These models were further integrated with three different nature-inspired metaheuristic algorithms—Particle Swarm Optimization (PSO), Genetic Algorithm (GA), and Simulated Annealing Harmony Search (SAHS)—to determine the optimal process parameters. Similarly, Cao et al. [25] focused on surface roughness and dimensional accuracy as response variables for LPBF parts and developed a Kriging model to quantitatively describe the relationship between key process parameters and these response variables. The model was then coupled with the Whale Optimization Algorithm (WOA) to optimize the process parameters, resulting in improved surface roughness and dimensional accuracy. Zhang et al. [26] combined artificial neural networks with fuzzy logic to create an Adaptive Neuro-Fuzzy Inference System (ANFIS) for predicting the fatigue life of 316L stainless steel samples fabricated via LPBF. Their model accurately predicted the fracture mechanisms and fatigue life of specimens based on different process and post-processing parameters. Mehrpouya et al. [27] developed a prediction model for process parameter optimization based on an artificial neural network to tailor the functional and mechanical behavior of printed NiTi parts, especially mechanical properties such as strain recovery rate and transition temperature. Additionally, Vijayaraghavan et al. [28] proposed an Improved Multi-Gene Genetic Programming (Im-MGGP) method to model the functional relationship between wear strength and input process variables in Fused Deposition Modeling (FDM). The results demonstrated that the improved method outperformed traditional MGGP, Support Vector Regression (SVR), and ANN models, providing better generalization for predicting the wear strength of FDM-fabricated parts. These above studies show that the optimization of process parameters based on statistical models and machine learning techniques is a time-saving and efficient method, which does not require any physics-based equations and only requires the use of past experimental data to establish the relationship between input variables and output targets. Additionally, they enable the exploration of appropriate process windows, avoiding the need for labor-intensive trial-and-error experiments.

However, existing studies on the relationship between process parameters and balling defects primarily rely on single-factor analysis involving numerous single-track experiments and typically employ subjective evaluations to assess the “balling level” (a term referring to the severity of balling defects in this paper). The shortcomings brought by such approaches are that the interactive effect of various parameters on balling defects remains insufficiently analyzed, and the subjective evaluation of balling level often leads to inaccuracies in determining the optimal combination of process parameters. To our knowledge, there has been no systematic investigation utilizing statistical models or machine learning techniques to explore the relationship between process parameters and the balling level, nor have there been reports on process parameter optimization specifically targeting the balling level. Therefore, this study presents a data-driven framework to predict and optimize the balling level on the interlayer surface of the parts during the LPBF process. Within this framework, machine learning techniques are employed to predict the balling level on the interlayer surfaces under different combinations of process parameters, while an improved genetic algorithm is then used to search for globally optimal process parameters. Finally, the optimization results are validated through a confirmation experiment. There are three main innovations in this work. First, balling features are extracted using a fully convolutional network (FCN), enabling a quantitative assessment of the balling level. Second, the balling level is selected as the response variable for LPBF components, and the relationship between the key process parameters and the balling level is established by the models of polynomial regression (PR), support vector regression (SVR), and backpropagation neural network (BPNN). Then, A comparative analysis of the prediction accuracy of these models is conducted to identify the optimal prediction model. Third, an improved genetic algorithm (IGA) is employed to optimize the process parameters, resulting in the attainment of optimal balling levels on the interlayer surface of the part. The results demonstrate that the proposed data-driven model is both feasible and effective in optimizing LPBF process parameters. This approach enables the attainment of an ideal balling level on the specimen’s interlayer surface, providing a practical and quantitative method to mitigate balling defects and enhance fabrication quality in the LPBF process.

The remainder of this paper is organized as follows: Section 2 details the experimental arrangements. Section 3 introduces the methodology of the proposed data-driven framework. In Section 4, the performance of various predictive and optimization models is evaluated, and the optimal process parameters are validated. Finally, Section 5 outlines the conclusions.

## 2. Experimental Study

### 2.1. LPBF System and Materials

**Figure 1:**
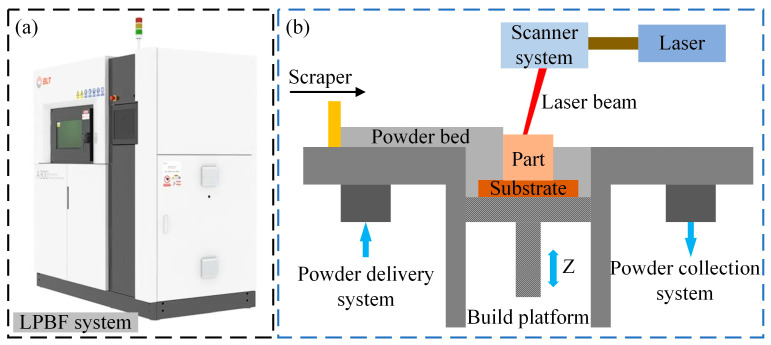
The setup and the working principle of the LPBF system: (**a**) The LPBF machine; (**b**) The schematic of the LPBF system work principle [25].

As shown in Figure 1, the LPBF system platform and its working principle are illustrated. The build chamber has dimensions of 250 mm × 250 mm × 300 mm. The system (BLT-A300, Xi’an Bright Laser Technologies Co., Ltd. (BLT) (Xi’an, China)) primarily consists of a fiber laser, a scanning galvanometer, a powder feeder, preheating components, and control modules. The fiber laser operates in continuous mode, with a wavelength range of 1060 nm to 1080 nm and a maximum output power of 500 W. The scanning speed ranges from 0 to 7 m/s, and argon is used as the shielding gas. During the manufacturing process, the powder feeder first deposits a thin layer of powder with a specified thickness onto the substrate. Subsequently, the high-energy fiber laser beam, guided by an F-theta scanning lens, selectively melts the metal powder according to the sliced files of the part’s 3D CAD model. Once the molten layer cools and solidifies, the building platform moves downward, and the powder delivery system moves upward to reach a predefined thickness. The scraper then spreads a new layer of powder over the solidified layer to begin the next layer fabrication. This process is repeated layer by layer until the part is fully built.

The powder material used in this experiment was commercially available 316L stainless steel powder with particle sizes ranging from 15 um to 45 um. To eliminate the potential influence of material differences between the substrate and the metal powder on the experimental results, the substrate was also made of 316L stainless steel. During the fabrication of cuboid specimens with dimensions of 10 mm × 10 mm × 1 mm, a stereomicroscope (ZXL-300, Shanghai Zhengxi Instrument Equipment Co., Ltd. (Shanghai, China)) equipped with a continuously variable magnification monocular microscope and a CCD camera, was employed to capture microscopic images of the specimens’ interlayer surfaces at 400× magnification and a resolution of 2592 × 1944 pixels. These images provided sufficient information on the balling defects occurring on the interlayer surfaces, thus providing a basis for subsequent quantitative evaluations of balling levels.

### 2.2. Evaluation of Balling Levels

**Figure 2:**
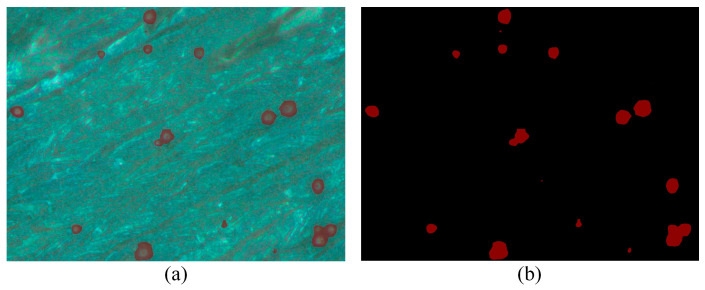
Balling feature extraction results of the FCN model: (**a**) Visualization image; (**b**) Label image.

Balling levels refer to a measure of the number and size of balling particles on the forming layer during the LPBF process, which directly reflects the severity of balling defects. To quantitatively evaluate the balling levels of the formed specimens under different process parameters, this section employs a fully convolutional neural network (FCN) to extract balling features from microscopic images captured from the formed specimen’s interlayer surface. These extracted features are then used to perform a quantitative assessment of the balling levels. Unlike conventional convolutional neural networks (CNNs), the FCN replaces the last fully connected layer with a convolutional layer, enabling it to train as an end-to-end and point-to-point network for pixel-level segmentation [29]. After training and optimizing the FCN model, the balling features were effectively separated from the complex background, with the final segmentation results presented in Figure 2.

From the feature extraction visualization image in Figure 2a, nearly all metallic balling particles are extracted from the formed specimen’s surface. Figure 2b shows the corresponding label diagram of the feature extraction results, where the balling feature areas are marked in red (pixel value is 1), and the background is shown in black (pixel value is 0). Then, the balling level on the surface of a single specimen can be evaluated using a metric called balling rate (Br), which is accurately calculated by Equation (1).

(1) $$Br=\frac{1}{N}\sum_{k=1}^{N}(\frac{\sum_{i=1}^{H}\sum_{j=1}^{W}P_{ijk}}{H\cdot W})$$

where: Br is the balling rate on the surface of a formed specimen, $P_{ijk}$ is the pixel value at the position (*i*, *j*) in the *k*-th microscopic image, *H* and *W* are the height and width of the collected microscopic image, respectively, and *N* is the number of uniformly collected microscopic images on the surface of a single specimen.

### 2.3. Experimental Design and Results

To construct the predictive models, full-factorial experiments involving three factors were conducted. The factors included laser power (P), scanning speed (V), and hatch space (H), which are key process parameters influencing the balling phenomenon in LPBF. As shown in Table 1, each factor was set at three different levels in this experimental design. Table 2 presents the experimental parameter settings and corresponding results. A total of 27 groups of sample data were fabricated, which later served as training samples for building the predictive models. In addition to these, five supplementary experiments with randomly designed process parameter combinations were performed to serve as test samples for evaluating the prediction accuracy of the constructed models. The parameter settings and results for these test experiments are provided in Table 3. For all experiments, the layer thickness was fixed at 0.05 mm, and the balling rate of each sample was measured using the balling level evaluation method, as described in the previous section.

## 3. Prediction and Optimization Methodologies

### 3.1. Overall Framework

**Figure 3:**
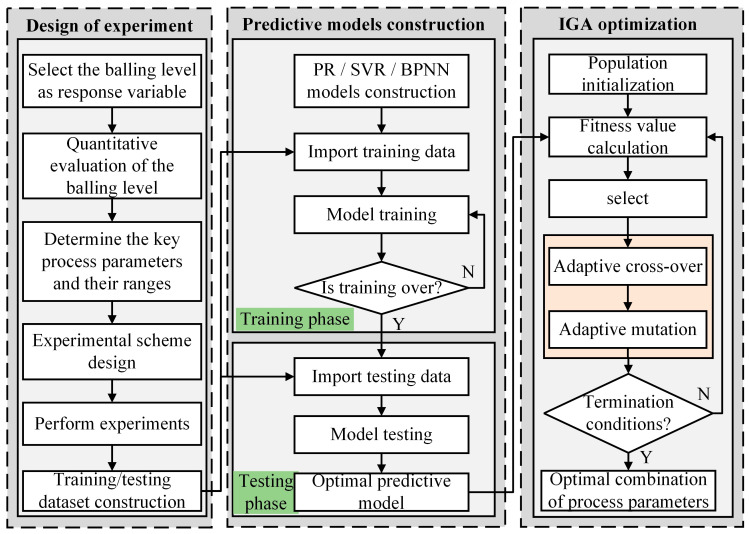
The data-driven framework for prediction and optimization of the balling levels on the interlayer surface of LPBF parts.

In this work, a data-driven framework integrating predictive models (including PR, SVR, BPNN) with an improved genetic algorithm (IGA) was developed to predict balling levels and identify optimal process parameters to achieve the desired surface quality of the formed specimens with minimal balling levels. The framework of the integrated approach is illustrated in Figure 3 and primarily consists of three stages: The design of the experiment (DOE), the construction of predictive models, and the optimization of process parameters based on IGA. The detailed descriptions of each stage are as follows:

(1) Design of experiment: First, the response variable and the design factor variables (P, V, and H), along with their respective ranges, are defined. The full-factorial method is then selected as the DOE method to generate various combinations of process parameters for the training sample points. Additionally, five supplementary experiments with randomly designed process parameter combinations are designed to serve as test sample points. Finally, the corresponding experiments are performed using the LPBF system.

(2) Construction of predictive models: Based on the experimental data obtained in Section 2.3, 27 groups of sample data were used as training samples to construct three models for predicting the balling levels, namely PR, SVR, and BPNN. Subsequently, these three models were compared and tested using five additional groups of test samples, and the best-performing model was then selected as the optimal prediction model for subsequent process parameter optimization.

(3) Optimization of process parameters based on IGA: In the optimization of process parameters using the IGA, the previously selected optimal predictive model is used as the fitness function to evaluate the fitness value of each individual, and then the genetic evolution of the population is carried out through selection, adaptive crossover, and mutation operations to realize the search for the optimal combination of process parameters. Finally, the effectiveness of the optimized process parameters is verified by a confirmation experiment.

### 3.2. Construction of Models for Balling Levels Prediction

The predictive model aims to construct a model as close to the real physical space as possible through a certain amount of data, including input values and corresponding output response values. Its purpose is to address the issue of excessive data demand in the process of predicting the balling levels and optimizing process parameters. In this section, three models, including PR, SVM, and BPNN, were developed to model the relationship between process parameters and the balling levels.

#### 3.2.1. Polynomial Regression

In a typical multi-input and single-response experiment, the method of PR models the response as a function of all primary factors and their interaction effects. For models of LPBF with multiple interactions between the primary input variables, a second-order polynomial function is adopted in this work. The corresponding formula is expressed as follows [30]:

(2) $$y=\alpha_{0}+\sum_{n}\alpha_{n}X_{n}+\sum_{n}\alpha_{n(n+1)}X_{n}X_{n+1}+\sum_{n}\alpha_{nn}X_{n}^{2},n=1,2,3$$

where *y* represents the predicted value of the balling rate; *n* is the number of input variables; $X_{1}$, $X_{2}$, $X_{3}$ represent laser power, scanning speed, and hatch space, respectively. $\alpha_{0}$ is a constant term; $\alpha_{n}$, $\alpha_{n(n+1)}$, $\alpha_{nn}$ and are linear coefficients, interaction coefficients, and quadratic coefficients, respectively.

#### 3.2.2. Support Vector Regression

Similar to the Support Vector Machine (SVM) used for classification, Support Vector Regression (SVR) also aims to find an optimal hyperplane in the data points; however, in regression problems, this hyperplane represents a regression function that is as close as possible to the data points while ensuring the predictions within a specified margin of allowable error [31]. For the LPBF process parameters *x* and the output balling rate *y*, the SVR model can be expressed as [32]:

(3) f(x)=w⋅ϕ(x)+b

where: ϕ(x) represents the nonlinear mapping of the input sample space to the feature space; w denotes the weight coefficients; and *b* is the bias term. According to the principle of structural risk minimization, the regression problem for the above function can be addressed by solving the parameters w and *b* through the following optimization problem [32].

(4) $$\left\{\begin{array}{c} \min\frac{1}{2}∥w{∥}^{2}+C\sum_{i=1}^{l}\left(\xi_{i}-\xi_{i}^{*}\right)\\ s.t.\;\left\{\begin{array}{c} \;y_{i}-w\cdot \phi \left(x_{i}\right)-b⩽\varepsilon +\xi_{i}\\ w\cdot \phi \left(x_{i}\right)+b-y_{i}⩽\varepsilon +\xi_{i}^{*}\\ \xi_{i}⩾0,\;\xi_{i}^{*}⩾0,\;i=1,2,\cdots ,l \end{array}\right. \end{array}\right.$$

where *l* represents the number of data points; *C* is the regularization coefficient; ε is the insensitivity coefficient; $\xi_{i}$ and $\xi_{i}^{*}$ are the slack variables; and $\frac{1}{2}{∥w∥}^{2}$ represents the confidence risk of the model, which is used to control the model’s complexity. By solving the above equations, the solved SVR model can be expressed as [32]:

(5) $$f(x)=w\cdot \phi (x)+b=\sum_{\mathrm{SV}}\left(a_{i}^{*}-a_{i}\right)\cdot K\left(x_{i},x\right)+b$$

where: $a_{i}^{*}$ and $a_{i}$ are the Lagrange multipliers; $K\left(x_{i},x\right)$ represents the kernel function; $x_{i}$ denotes the process parameter combination of *i*-th data point in this work.

In the SVR model, the commonly used kernel functions include the following three types [33,34]:

Polynomial Kernel:

(6) $$K\left(x_{i},x\right)={\left[\gamma \left(x_{i}\cdot x\right)+\alpha_{\mathrm{coef}}\right]}^{d}$$

where: γ is the scaling factor; $\alpha_{\mathrm{coef}}$ is a constant term; d is the degree of the polynomial.

Sigmoid kernel:

(7) $$K\left(x_{i},x\right)=\tanh\left[\gamma \left(x_{i}\cdot x\right)+\alpha_{\mathrm{coef}}\right]$$

where: γ is the scaling factor; $\alpha_{\mathrm{coef}}$ is a constant controlling the offset.

Radial Basis Function (RBF) Kernel:

(8) $$K\left(x_{i},x\right)=\exp\left(-\gamma {\left\|x_{i}-x\right\|}^{2}\right)$$

where: γ is a kernel parameter that controls the smoothness of the function.

All three kernel functions will be tested to determine the optimal SVR model with the best performance for predicting balling levels, as discussed later in Section 4.1.2.

#### 3.2.3. Back Propagation Neural Network

**Figure 4:**
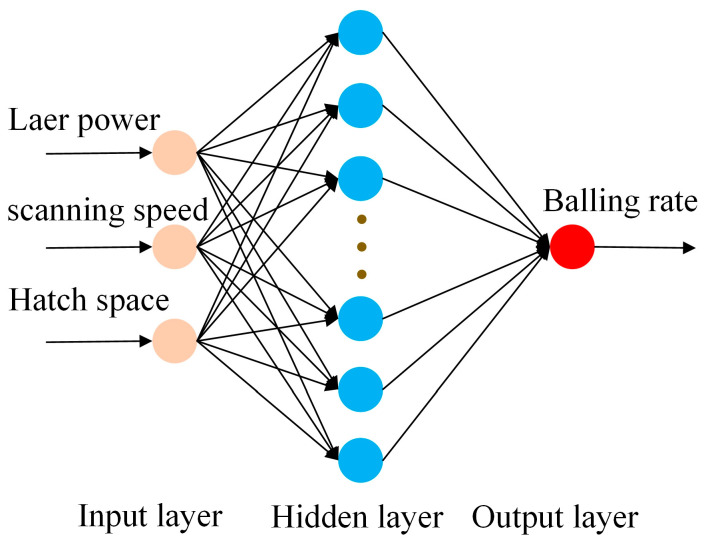
Topological structure of the BPNN model.

BPNN is a network inspired by the structure and function of biological neural networks. It utilizes the error backpropagation algorithms to learn from input/output training samples provided externally. By iteratively adjusting the connection weights of the network based on the error signal, the predictive output gradually approximates the desired target output. This method offers incomparable advantages in dealing with highly nonlinear problems. Considering the complex nonlinear coupling relationship between process parameters and the balling rate in LPBF, this section aims to establish a three-layer BPNN model with the process parameters as input and the balling rate as output for comparison. The topological structure of the network is illustrated in Figure 4.

As can be seen from Figure 4, the BPNN model also contains a hidden layer in addition to the input and output layers. Generally, the number of nodes in the hidden layer significantly affects the network’s performance. Therefore, this study adopts the following empirical formula [35] to determine the number of nodes in the hidden layer:

(9) $$l=\sqrt{n+m}+b$$

where *n*, *l*, and *m* represent the number of nodes in the input layer, hidden layer, and output layer, respectively, and *b* is a constant ranging from 1 to 10.

### 3.3. Genetic Algorithm and Its Improved Method

#### 3.3.1. Standard Genetic Algorithm

The genetic algorithm (GA) is a population-based stochastic algorithm inspired by Darwin’s theory of evolution and Mendelian genetics [24,36]. It mimics the mechanisms of biological evolution and functions as an efficient, parallel, and global search method. As illustrated in Figure 3, the optimization process using a genetic algorithm begins with the creation of a randomly generated initial population, which represents candidate solutions for the problem to be solved. Subsequently, a selection operator identifies individuals with high fitness values within the current population to form a parent population, and then new offspring individuals are generated through crossover and mutation operations. This iterative process continues, progressively optimizing the population until a predefined termination condition is satisfied. Notably, during each iteration of the genetic algorithm, the fitness value of each individual is evaluated using the optimal predictive model obtained in the preceding stages.

#### 3.3.2. Improved Genetic Algorithm

Although the standard genetic algorithm offers advantages such as global search capability, inherent parallelism, and scalability, it also has certain limitations. Specifically, the algorithm exhibits poor local search ability, reduced search efficiency in the later stages of evolution, and a tendency toward premature convergence. Therefore, it is crucial to carefully select and design the parameters that impact both the convergence properties and the convergence speed of the algorithm in practical applications. The improved genetic algorithm proposed in this section incorporates the following features.

(1) On the problem of fitness function selection: For the fitness value of population individuals, one of the most intuitive approaches is to directly use the reciprocal of the predicted value from the optimal predictive model as the fitness value. According to the 27 groups of sample data presented in Section 2.3, the balling rate obtained for different combinations of process parameters falls within the range of 0.751 to 2.807. Correspondingly, the range of fitness values is 0.3 to 1.4. This narrow range indicates that, despite significant variations in the process parameters, the changes in fitness values are relatively small. As a result, when the reciprocal of the predicted value is directly applied as the fitness value for individuals, the genetic algorithm becomes less sensitive to changes in process parameters during the iterative optimization process, leading to slow convergence and a tendency to converge to a local optimum. To address these issues, the fitness function is adjusted as follows:

(10) F=10⋅(φ−0.3)

where *F* represents the fitness value of an individual and φ is the reciprocal of the predicted value obtained from the optimal predictive model for different combinations of process parameters. In this case, the range of fitness values extends to 0~11. Compared to the original fitness value range before adjustment, the expanded range enhances the sensitivity of the genetic algorithm to changes in process parameters during the iterative optimization process.

(2) Regarding the improvement of prematurity in GA: A novel metric is introduced to evaluate the degree of prematurity in a population. The primary manifestation of prematurity in a population is the repetition or homoplasy of some of the fittest individuals within the population. This phenomenon leads to a high probability of these individuals being selected and reproduced in subsequent generations. As a result, the offspring produced through the crossover of these individuals exhibit minimal variation from their parents, causing the optimization process of the genetic algorithm to slow significantly and reducing its overall search efficiency. Therefore, accurately assessing whether a population is experiencing prematurity primarily depends on whether the current fittest individuals in the population are repetitive and homoplasy. Based on this concept, a new metric, termed the homoplasy degree of superior individuals (denoted as ∆), is proposed to evaluate the degree of prematurity in a population. The metric is defined as follows:

Suppose that the population in generation *t* consists of *M* individuals, with an average fitness value denoted as $\bar{F_{t}}$, and the optimal individual fitness value as $F_{t,\max}$. Among these individuals, there are *N* individuals whose fitness values exceed $\bar{F_{t}}$, and their respective fitness values are denoted as $F_{t,n}$. Furthermore, $\bar{F_{t,\max}}$ represents the average fitness value of those individuals whose fitness values are greater than $\bar{F_{t}}$. Thus:

(11) $$\Delta =\frac{1}{N-1}\sum_{n=1}^{N}{\left(F_{t,n}-\bar{F_{t,\max}}\right)}^{2}$$

The metric ∆ represents the fitness value variance among individuals in the population whose fitness values exceed the average fitness value ($\bar{F_{t}}$) of the population. It is designed to characterize the degree of prematurity within the population. During its calculation, individuals with fitness values below the average fitness are excluded, thereby avoiding the adverse effects caused by lower-fitness individuals. This exclusion enables a clearer conceptual reflection of the homoplasy degree among the fittest individuals in the population, allowing for a more accurate description of the prematurity that occurs within the population.

(3) Adaptive crossover and mutation: Controlling genetic evolution using fixed control parameters can easily result in prematurity and reduce the search efficiency of the algorithm. Currently, one of the most effective approaches to adjust the control parameters in GA is dynamic adaptive technology. The fundamental principle of this technique is to adaptively adjust the crossover probability ($P_{c}$) and mutation probability ($P_{m}$) based on the actual state of the population during the evolutionary process. Specifically, when the population tends to converge, $P_{c}$ is reduced and $P_{m}$ is increased to maintain population diversity and avoid prematurity. Conversely, when the population individuals are highly divergent, $P_{c}$ is increased and $P_{m}$ is reduced, guiding the population toward convergence and improving the convergence speed of the algorithm.

Based on the previously introduced population prematurity evaluation metric ∆, a new adaptive strategy for adjusting the control parameters of GA is proposed. This strategy enables the crossover probability $P_{c}$ and mutation probability $P_{m}$ to dynamically vary during the evolutionary process in response to the changes ∆. The specific mathematical description is as follows:

(12) $$P_{c}=\frac{1}{1+k_{1}\cdot \exp(-\Delta )}$$

(13) $$P_{m}=1-\frac{1}{1+k_{2}\cdot \exp(-\Delta )}$$

where: $k_{1}$ and $k_{2}$ are real-valued parameters, both greater than zero, used to control the range of $P_{c}$ and $P_{m}$. From the above equations, it can be observed that $P_{c}$ and $P_{m}$ dynamically and adaptively adjust according to the value ∆ throughout the evolutionary process. Specifically, when the population individuals tend to diverge (indicated by a larger value of ∆), $P_{c}$ it increases while $P_{m}$ decreasing, enhancing the population’s ability to develop superior individuals. Conversely, when the population individuals tend to converge (indicated by a smaller value of ∆), $P_{c}$ it decreases while $P_{m}$ increasing, improving the population’s ability to generate new individuals and maintain diversity.

## 4. Results and Discussion

### 4.1. Models Prediction Results

#### 4.1.1. Prediction Results of the Polynomial Regression Model

**Figure 5:**
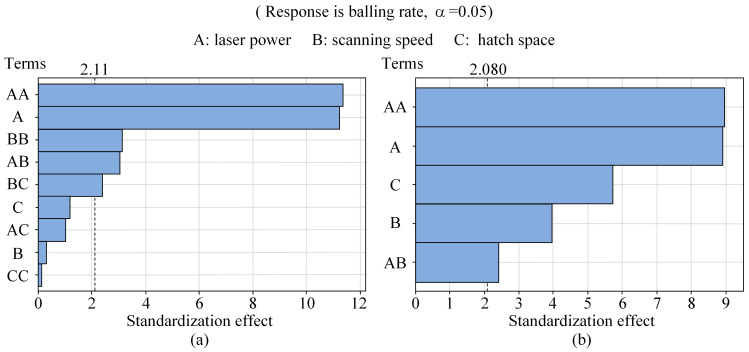
Pareto diagram before and after optimization of the polynomial regression model: (**a**) before optimization; (**b**) after optimization.

With the help of Minitab software (version number is 21.1.0.0), a second-order polynomial regression analysis was performed on the experimental data presented in Table 2. Simultaneously, an analysis of variance (ANOVA) was utilized to determine the significance of each term in the regression polynomial, thereby eliminating insignificant terms during the equation fitting process. Figure 5a presents the Pareto diagram of the model considering all terms of the second-order polynomial. It can be observed that the terms *A*, *AB*, *BC*, *A*^2^, and *B*^2^ were significant, whereas *B*, *C*, *AC*, and *C*^2^ were insignificant. It is worth noting that previous research has already demonstrated that the main effects of these three factors significantly influence the balling defects [21,37,38,39]. Therefore, this initial model fails to accurately represent the correlation between the balling rate and the process parameters. To achieve a more precise model and results, manual optimization of the model was conducted. This process involved sequentially removing the insignificant terms and performing regression fitting. The Pareto diagram of the optimized model is shown in Figure 5b, where *A*, *B*, *C*, *AB*, and *A*^2^ are identified as significant terms. Table 4 presents the ANOVA results for the optimized model, and the corresponding regression equation for the balling rate is as follows:

(14) $$Br=20.03-0.2478A+0.01957B-3.820C+0.000828A^{2}-0.000079AB$$

where *Br* represents the predicted balling rate, while *A*, *B*, and *C* denote the process parameters of laser power, scanning speed, and hatch space, respectively.

**Figure 6:**
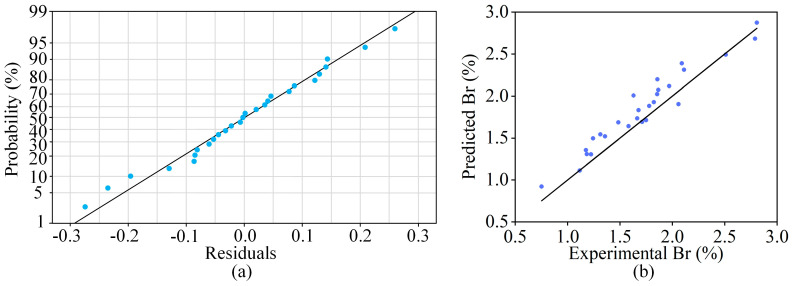
Effectiveness validation of polynomial regression model: (**a**) Residual probability distribution; (**b**) Comparison between the experimental and predicted values of balling rate.

To validate the effectiveness of the optimized regression model, residual analysis and the distribution patterns of the data points were utilized to evaluate the regression equation. Figure 6a shows the residual probability distribution plot, where all points are arranged almost linearly along a straight line, indicating that the residuals approximately follow a normal distribution. By comparing the predicted values of the balling rate with the experimental values through the fitted curve (as shown in Figure 6b), the errors between them were found to be small. Furthermore, the points in the plot are distributed roughly along a straight line, confirming that the model’s prediction results align well with the experimental results. Thus, the regression equation demonstrates a high degree of accuracy.

#### 4.1.2. Prediction Results of the Support Vector Regression Model

**Figure 7:**
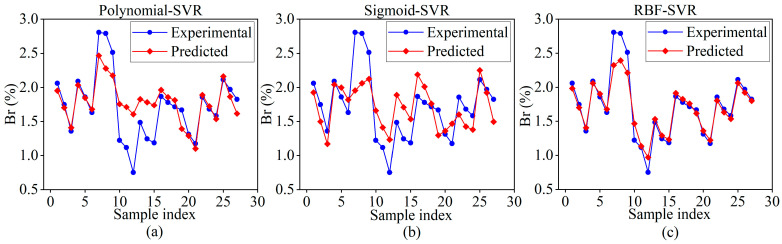
Prediction results of SVR models with different kernel functions: (**a**) Polynomial, (**b**) Sigmoid, (**c**) RBF.

In this section, SVR models with three different kernel functions (e.g., sigmoid, polynomial, radial basis function (RBF)) were utilized to model the LPBF process, and their predictive performances were evaluated individually. Figure 7 illustrates the prediction results of these SVR models, which were not yet optimized for hyperparameters, on the training samples.

The predictive performance of each model was evaluated using the metrics of mean absolute error (MAE), mean square error (MSE), and the coefficient of determination (R^2^), with their comparison results presented in Table 5. As shown in the table, for different kernel functions, the ranking of MAE and MSE values, from largest to smallest, is as follows: Sigmoid > Polynomial > RBF, while the ranking of R^2^ values, from largest to smallest, is RBF > Polynomial > Sigmoid. These findings indicate that the SVR model with the RBF kernel function outperforms the others across various evaluation metrics, demonstrating superior adaptability to small sample sizes and producing more accurate predictions of the balling rate. Therefore, the RBF kernel function was selected for the SVR model in this study.

**Figure 8:**
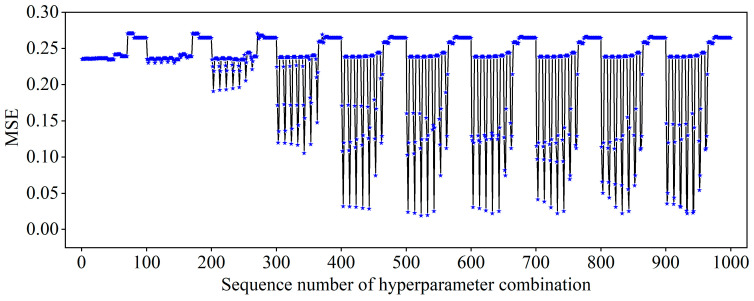
Variation of the MSE in the SVR model under different hyperparameter combinations.

To obtain the optimal SVR model, the penalty factor *C*, the insensitivity coefficient ε, and the kernel function parameter γ must be optimized during model training. The penalty factor *C* determines the trade-off between minimizing the fitting error and controlling the complexity of the model. The insensitivity coefficient ε specifies the tolerance for errors within which deviations from the actual values are not penalized, while the kernel parameter γ governs the nonlinear mapping from the input space to the high-dimensional feature space. To ensure reliable hyperparameter optimization, a 5-fold cross-validation combined with a grid search method was employed. Specifically, the search library for *C* was set to [0.001, 0.01, 0.1, 1, 10, 20, 50, 100, 200, 500], the search library for ε was [0.01, 0.02, 0.05, 0.1, 0.2, 0.5, 1, 2, 5, 10], and the search library for γ was [0.001, 0.01, 0.1, 1, 10, 20, 50, 100, 200, 500]. This resulted in a total of 1000 parameter combinations. Taking 27 groups of sample data obtained from the experiment as training samples, the variation in MSE of the SVR model on the validation dataset under different parameter combinations is shown in Figure 8.

**Figure 9:**
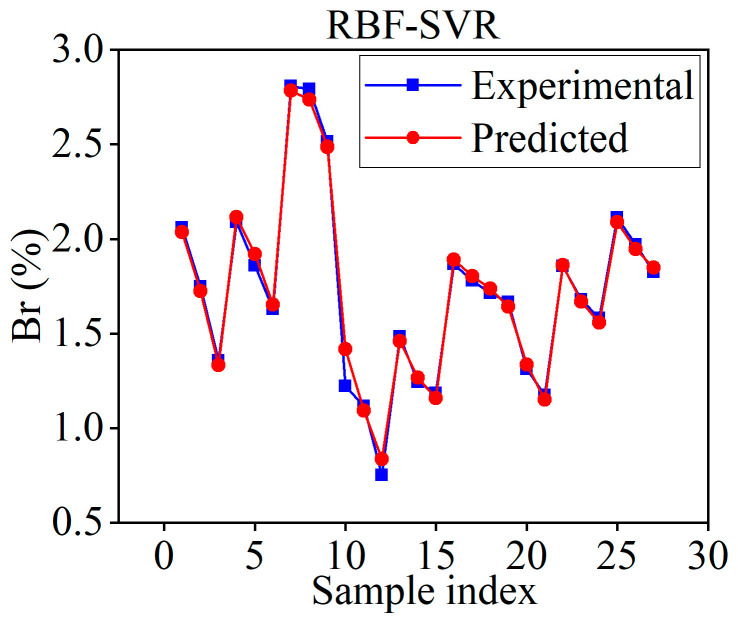
Prediction results of the optimized RBF-SVR model.

As can be seen from the figure above, when the parameter combination traverses to the 523rd group, the MSE of the SVR model on the validation dataset is minimized to 0.0191. This indicates that the 523rd parameter combination is the optimal one, with the corresponding parameter values being *C* = 20, ε = 0.05, γ = 0.1. These optimized parameter values were then incorporated into the SVR model, which was retrained using the processed sample data. After completing the training, the training samples were used again as the input to test the accuracy of the model. Figure 9 presents the prediction results of the optimized RBF-SVR model on the training samples. At this stage, the evaluation metrics of the model on the training samples are as follows: MAE = 0.0339, MSE = 0.0024, R^2^ = 0.9898.

#### 4.1.3. Prediction Results of the Back Propagation Neural Network Model

**Figure 10:**
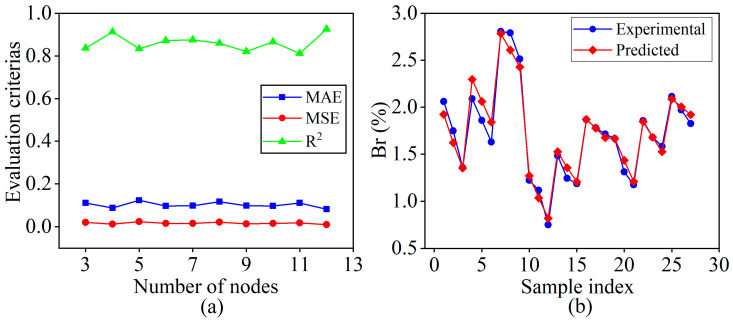
BPNN: (**a**) Performance comparison of the BPNN model with different numbers of nodes in the hidden layer; (**b**) Prediction results of the optimal BPNN model.

In this work, the input and output nodes of the BPNN model are *n* = 3 and *m* = 1, respectively. Based on the empirical Formula (9), the number of hidden layer nodes can be calculated as 3 ≤ *l* ≤ 12. To determine the optimal topological structure of the BPNN model with the best performance, BPNN models with different numbers of hidden layer nodes were evaluated using a 5-fold cross-validation experiment on the training samples. As shown in Figure 10a, the performance comparison on the validation dataset indicates that when the number of hidden layer nodes *l* = 12, the network achieves the smallest MAE and MSE, as well as the highest R^2^. Therefore, the BPNN with 12 hidden layer nodes was selected as the final topological structure for the model used in this work.

Figure 10b shows the prediction results of the trained optimal BPNN model on the training sample dataset. It can be observed that, for different combinations of process parameters, the predicted values of the balling rate are in good agreement with the experimental values. At this stage, the model achieves a MAE of 0.0731, a MSE of 0.0097, and a R^2^ of 0.9579 on the training sample dataset.

### 4.2. Comparison of Prediction Performance Among These Three Models

To select the model with the best fitting performance for subsequent process parameter optimization, the above three models (PR, SVR, and BPNN) were compared and analyzed in this section. Specifically, the five groups of test samples listed in Table 3 were used as inputs for the three trained models. The predicted values of the balling rate produced by these models were then compared with the corresponding experimental values to evaluate the generalization ability of each model. Table 6 presents the prediction results of the five groups of test samples obtained from these three models.

**Figure 11:**
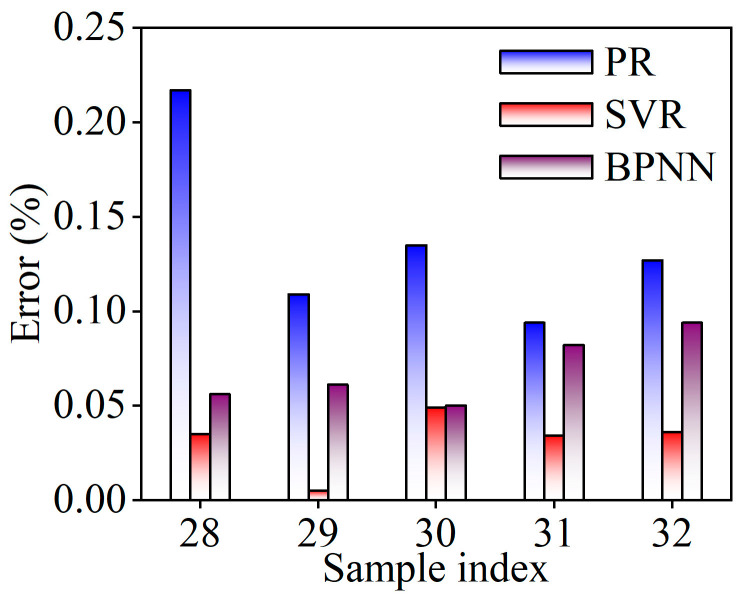
Comparison of prediction errors of balling rate for different models.

The prediction errors of the balling rate for these three models are compared in Figure 11. It can be observed that the SVR model demonstrates significantly higher prediction accuracy compared to both the PR and BPNN models. This indicates that the SVR model is better suited for constructing a quantitative mathematical relationship between process parameters and the balling rate. Therefore, the subsequent optimization of process parameters will be conducted based on the SVR model, which exhibits minimal error.

### 4.3. Optimization of Process Parameters Based on IGA

#### 4.3.1. Parameter Settings of Genetic Algorithms and Their Optimization Results

In the improved genetic algorithm, the encoding mode of the process parameters is real-number encoding, and the three process parameters of laser power (P), scanning speed (V), and hatch space (H) are sequentially taken as gene to form a chromosome, with the form U = [P, V, H]. Each chromosome represents a combination of process parameters, and the real value of each gene in the chromosome corresponds to the value of a specific process parameter. Considering the practical problems to be solved, it is necessary to set constraints for each gene, that is, to define constraint intervals for different process parameters. Based on the full factorial experimental factor-level design described in Section 2.3, the ranges for the process parameters are specified, as shown in Table 7.

**Figure 12:**
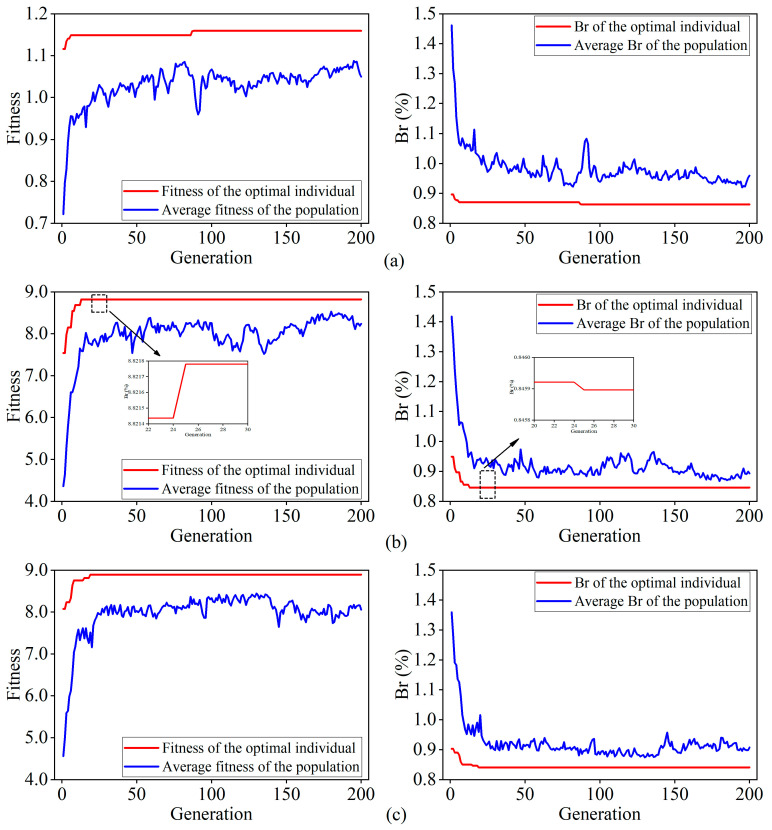
Variation curves of fitness and balling rate of different genetic algorithms: (**a**) B-GA; (**b**) FFI-GA; (**c**) A-GA.

To optimize the process parameters and verify the effectiveness of the improved genetic algorithm, comparative experiments were conducted using three different methods: the Basic Genetic Algorithm (B-GA), the Fitness Function Improved Genetic Algorithm (FFI-GA), and the Adaptive Genetic Algorithm (A-GA). Among them, the B-GA refers to the standard genetic algorithm without any improvements. During the algorithm’s iterations, the fitness values of individuals are defined as the inverses of the predicted values obtained from the SVR model, and both the crossover and mutation probabilities are set as fixed values. The FFI-GA is a standard genetic algorithm that incorporates an improved fitness function, as described in Section 3.3.2, but without further changes to the crossover or mutation probabilities. The A-GA, proposed in this study, introduces adaptive crossover and mutation probabilities based on the FFI-GA to further enhance optimization performance. All three genetic algorithms employed the roulette wheel selection method as the selection operator, with an initial population size of *M* = 50 and a maximum evolutionary generation of *G* = 200. For B-GA and FFI-GA, the crossover probability $P_{c}$ is fixed at 0.4, and the mutation probability $P_{m}$ is fixed at 0.09. For the A-GA, the real-valued parameters $k_{1}=1.5$ and $k_{2}=0.1$, resulting in a crossover probability $P_{c}$ ranging from 0.4 to 1 and a mutation probability $P_{m}$ ranging from 0 to 0.09. Figure 12 shows the curves of average population fitness, optimal individual fitness, average population balling rate, and optimal individual balling rate in the optimization process of these three algorithms. The final optimized process parameter results for each algorithm are summarized in Table 8.

#### 4.3.2. Analysis of Optimization Results

As can be seen from Figure 12, with the progress of evolution, the average fitness value of the population in these three algorithms exhibits a stepwise increase, eventually stabilizing near the fitness value of the optimal individual. In contrast, the average balling rate of the population follows the opposite trend, displaying a stepwise decrease and gradually converging toward the balling rate of the optimal individual as the number of iterations increases. Both trends indicate that the evolutionary processes of these three algorithms are proceeding in the correct direction. As the iterations progress, the overall population evolves from the initial state, leading to the average fitness value and balling rate of the population increasingly approximating those of the optimal individual. This demonstrates that the application of genetic algorithms for process parameter optimization in LPBF is feasible and effective.

In combination with Table 8, it can be observed that the corresponding balling rate and the required number of iterations when the three genetic algorithms converge are in the order from smallest to largest: A-GA < FFI-GA < B-GA. This indicates that the adaptive genetic algorithm proposed in this study achieves the optimal process parameters and exhibits the fastest convergence speed. Given the differences in these three optimization results, the fundamental causes can be explained from the following aspects:

Firstly, the B-GA algorithm directly takes the reciprocal of the predicted values generated by the SVR model as the fitness value for individuals. Under this setting, the fitness value range is relatively small, leading to a lack of prominent differences in fitness among individuals. Consequently, when the roulette wheel selection method is employed, the probability of selecting superior individuals becomes insufficiently distinct. This, in turn, slows the propagation of high-quality genes within the population. Moreover, the small range of fitness values implies that there are multiple different gene combinations that may correspond to similar fitness values, thereby increasing the difficulty of identifying the optimal gene combination. Additionally, the use of fixed crossover and mutation probabilities hampers the algorithm’s ability to balance global and local search processes. This limitation reduces its capacity to fine-tune solutions near the optimum during the later stages of evolution. As a result, the algorithm’s convergence performance is negatively affected, making it prone to being trapped in a local optimum. As depicted in Figure 12a, although the B-GA algorithm approaches a local optimum by the 6th generation, the improvement in fitness value brought by evolution is minimal. With the continuous evolution process, the algorithm does not converge to the global optimum until the 88th generation, indicating a very slow convergence rate.

Secondly, compared to B-GA, the FFI-GA algorithm introduces a transformation of the fitness function to enhance the differentiation of fitness values. This adjustment improves the distinction of superior individuals during the evolutionary process. However, the FFI-GA algorithm still suffers from the limitations of fixed crossover and mutation probabilities, which restrict its capability to perform fine-grained local searches in the later stages of evolution. As illustrated in Figure 12b, the FFI-GA algorithm achieves faster convergence compared to B-GA, reaching convergence by the 25th generation. Furthermore, the optimal solution obtained at convergence is superior to that produced by the B-GA algorithm. Nevertheless, the solution remains a local optimum.

Finally, to address the challenge of balancing global and local search caused by fixed crossover and mutation probabilities, the A-GA algorithm proposed in this study introduces an adaptive mechanism for adjusting the crossover and mutation probabilities based on FFI-GA so that the algorithm can dynamically adapt its search strategy according to the evolutionary stage and realize self-regulated search intensity. As described in Section 3.3.2, when the population tends to converge, the crossover probability is reduced, and the mutation probability is increased to maintain population diversity and prevent prematurity. Conversely, when the population exhibits greater divergence, the crossover probability is increased, and the mutation probability is decreased to guide the population toward convergence. This approach can not only accelerate the convergence speed of the algorithm but also ensure that it converges to the global optimal solution. As shown in Figure 12c, the A-GA algorithm converges by the 19th generation, with a balling rate of 0.8409%. Compared with the other two algorithms, the A-GA algorithm demonstrates a significantly faster convergence speed, with the optimal individual at convergence corresponding to the lowest balling rate. These results further validate the effectiveness of the proposed A-GA algorithm.

**Figure 13:**
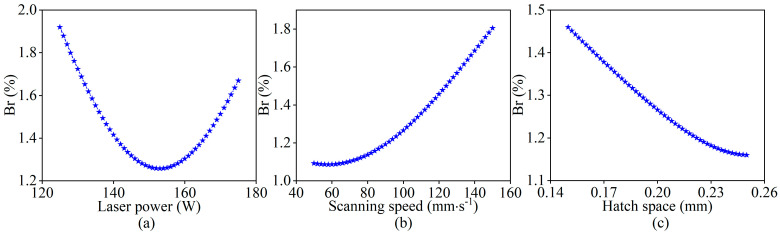
Influence of process parameters on the balling rate: (**a**) laser power, (**b**) scanning speed, (**c**) hatch space. Notably, when studying a certain process parameter, keep the middle value of other process parameters in the specified range and record the change of balling rate with the process parameter.

It is worth noting that the optimized process parameters obtained by these three genetic algorithms at convergence are almost identical in terms of laser power and hatch space, whereas the values for scanning speed differ significantly. Furthermore, as the scanning speed increases, the corresponding balling rate also increases, although the magnitude of this change is relatively small. This observation is consistent with the trend shown in Figure 13, where the balling rate increases slowly in the early stage but becomes more pronounced as the scanning speed continues to increase. All these indicate that there are multiple distinct gene combinations corresponding to similar fitness values during the algorithm’s evolutionary process, which increases the difficulty of identifying the optimal gene combination and also highlights the importance of enhancing the differentiation of fitness values to accelerate the evolutionary process of the algorithm and converge to the global optimal solution.

### 4.4. Validity Verification

**Figure 14:**
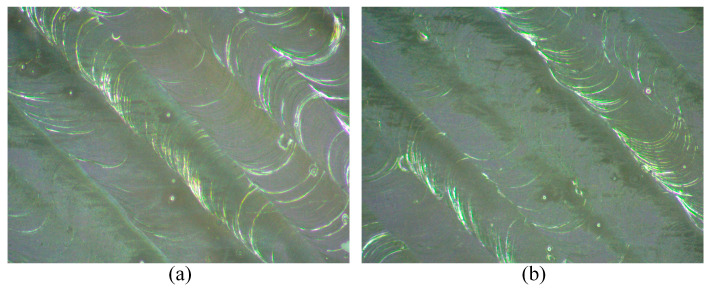
Microscopic images of the interlayer surface of the formed part under the process parameters combinations before and after optimization: (**a**) Before optimization; (**b**) After optimization.

To validate the effectiveness of the optimization results, practical printing experiments were conducted using the optimal process parameter combination obtained by the A-GA algorithm. Prior to the experiments, the current optimal process parameters were adjusted based on the characteristics of the LPBF equipment and the process parameter impact analysis presented in Figure 13. Specifically, the modified process parameters were as follows: laser power P = 153 W, scanning speed V = 50 mm/s, and hatch space H = 0.25 mm. These process parameters were subsequently employed in the printing experiments. Figure 14 presents a comparison of the interlayer surface micrographs of the specimens formed under the process parameter combinations before and after optimization. The process parameters prior to optimization correspond to the parameters at which the specimen achieved the minimum balling rate of 0.751% during the full-factorial experiment described in Section 2.3: P = 150 w, V = 50 mm/s, H = 0.25 mm. The comparison reveals that the specimens formed both before and after optimization exhibit smooth surfaces, continuous melt tracks, and good overlap between adjacent tracks. However, both also contain a small number of fine balling particles, making it challenging to visually distinguish differences in the balling levels. For a more precise comparison, the balling levels evaluation method described in Section 2.2 was employed to extract the balling features and calculate the balling rate of the specimen formed under the acquired optimal process parameter combinations. The result was then compared with that obtained prior to the optimization of process parameters. A detailed comparison of the results is provided in Table 9.

As shown in Table 9, compared with the process parameter combination before optimization, the balling rate predicted by the optimized process parameter combination decreased by 0.0061%, while the experimental value decreased by 0.005%. Although this reduction in the balling rate is minor, it demonstrates a measurable improvement in the balling level of the specimens. This result, on the one hand, confirms the reasonableness of the optimized process parameters and, on the other hand, validates the feasibility of combining SVR with GA for predicting balling levels and optimizing process parameters to control the balling level of a specimen. Furthermore, it highlights that adjusting process parameters is an effective strategy for minimizing the balling level on the interlayer surface of a specimen during the LPBF forming process.

## 5. Conclusions

To improve the interlayer surface quality of the specimens during the LPBF process, predictive models represented by PR, SVR, and BPNN were constructed to quantitatively evaluate the correlation between process parameters and the balling levels and the influence of each process parameter on balling levels was also analyzed. Furthermore, process parameter optimization was performed by combining the improved genetic algorithm with the acquired optimal predictive model, resulting in the identification of the optimal process parameter combination and its corresponding minimum balling level. Based on the experimental analysis and results, the following conclusions can be drawn:

- For modeling the correlation between process parameters and the balling level in LPBF, the SVR model demonstrates better fitting performance when compared with the models of PR and BPNN. This indicates that SVR is more suitable for constructing a quantitative mathematical model to represent the relationship between process parameters and the balling level.
- Within the specified range of process parameter values, the balling rate exhibits distinct variation trends in response to changes in different process parameters. Specifically, as laser power increases, the balling rate decreases initially and then increases, while it increases with the increase of scanning speed and decreases with the increase of hatch space.
- By combining the improved genetic algorithm with the support vector regression model, process parameter optimization was successfully achieved. The optimized process parameters were determined as P = 153 W, V = 50 mm/s, H = 0.25 mm. Compared to the process parameter combination before optimization, the predicted balling rate for the optimized process parameter combination was reduced by 0.0061%, while the experimental value decreased by 0.005%. These results indicate that the optimized process parameter combination is reasonable, and the integration of these two intelligent methods can effectively leverage their respective advantages, which is suitable for the optimization of the process parameters in the LPBF process.

Compared with the traditional methods, this study provides a quantitative approach to evaluating the severity of balling defects, allowing for a more precise analysis of how various process parameters influence balling levels. Additionally, the developed prediction and optimization models facilitate the identification of optimal process parameter windows and the best parameter combinations, thereby supporting the successful fabrication of high-quality components. In the future, research will focus on exploring the feasibility of layer-by-layer monitoring and control of forming quality related to balling defects, as well as the application of deep convolutional neural networks for layer-wise detection of balling levels during the LPBF process.

## Figures and Tables

**Table 1:** Process parameters of LPBF and their corresponding levels.

Factor	Units	Factor Level		
		1	2	3
Laser power (P)	W	125	150	175
Scanning speed (V)	mm/s	50	100	150
Hatch space (H)	mm	0.15	0.20	0.25

**Table 2:** Design of experiment for training samples and their corresponding results.

No.	P (W)	V (mm/s)	H (mm)	Br (%)	VED (J/mm^3^)
1	125	50	0.15	2.061	333.33
2	125	50	0.20	1.750	250.00
3	125	50	0.25	1.358	200.00
4	125	100	0.15	2.091	166.67
5	125	100	0.20	1.860	125.00
6	125	100	0.25	1.630	100.00
7	125	150	0.15	2.807	111.11
8	125	150	0.20	2.790	83.33
9	125	150	0.25	2.512	66.67
10	150	50	0.15	1.223	400.00
11	150	50	0.20	1.116	300.00
12	150	50	0.25	0.751	240.00
13	150	100	0.15	1.484	200.00
14	150	100	0.20	1.244	150.00
15	150	100	0.25	1.184	120.00
16	150	150	0.15	1.867	133.33
17	150	150	0.20	1.781	100.00
18	150	150	0.25	1.713	80.00
19	175	50	0.15	1.666	466.67
20	175	50	0.20	1.312	350.00
21	175	50	0.25	1.175	280.00
22	175	100	0.15	1.856	233.33
23	175	100	0.20	1.680	175.00
24	175	100	0.25	1.582	140.00
25	175	150	0.15	2.113	155.56
26	175	150	0.20	1.972	116.67
27	175	150	0.25	1.825	93.33

**Table 3:** Design of experiment for testing samples and their corresponding results.

No.	P (W)	V (mm/s)	H (mm)	Br (%)	VED (J/mm^3^)
28	135	90	0.21	1.450	142.86
29	145	70	0.17	1.317	243.70
30	155	130	0.19	1.605	125.51
31	165	110	0.23	1.432	130.43
32	135	70	0.17	1.514	226.89

**Table 4:** Results of variance analysis after optimization of polynomial regression model.

Source	*DF*	*Adj SS*	*Adj MS*	*F*-Value	*p*-Value
Regression	5	5.8287	1.16574	58.45	0.000
*A*—laser power	1	1.5845	1.58453	79.45	0.000
*B*—scanning speed	1	0.3134	0.31342	15.72	0.001
*C*—hatch space	1	0.6567	0.65666	32.93	0.000
*A* ^2^	1	1.6065	1.60649	80.55	0.000
*AB*	1	0.1166	0.11662	5.85	0.025
Error	21	0.4188	0.01994		
Total	26	6.2475			
Standard error	0.141219	*R* ^2^	0.9330	*R*^2^ (adjust)	0.9170

**Table 5:** Comparison of prediction performance of SVR models with different kernel functions.

Models	Metrics		
	MAE	MSE	R^2^
Polynomial-SVR	0.2245	0.1024	0.5574
Sigmoid-SVR	0.2923	0.1214	0.4753
RBF-SVR	0.0981	0.0234	0.8987

**Table 6:** Predicted values of balling rate from the three predictive models.

No.	Experimental Value of Br (%)	Predicted Value of Br (%)		
		PR	SVR	BPNN
28	1.450	1.667	1.415	1.394
29	1.317	1.426	1.322	1.256
30	1.605	1.740	1.556	1.655
31	1.432	1.526	1.398	1.514
32	1.514	1.641	1.550	1.420

**Table 7:** Constraint ranges of different process parameters.

Process Parameters	P (W)	V (mm/s)	H (mm)
Upper bound	125	50	0.15
Lower bound	175	150	0.25

**Table 8:** Comparison of optimization results of three different genetic algorithms for optimal process parameters.

Algorithms	Optimal Process Parameters			Br (%)	Number of Generations for Convergence
	P (W)	V (mm/s)	H (mm)		
B-GA	152.3780	58.0242	0.2453	0.8628	88
FFI-GA	152.4867	55.6764	0.2494	0.8458	25
A-GA	152.4887	51.4629	0.2466	0.8409	19

**Table 9:** Comparison of interlayer surface balling rate of specimens under the process parameters combinations before and after optimization.

Optimization State of Process Parameters	Process Parameters			Predicted Value of Br (%)	Experimental Value of Br (%)
	P (W)	V (mm/s)	H (mm)		
Before	150	50	0.25	0.8354	0.751
After	153	50	0.25	0.8293	0.746

## Data Availability

The original contributions presented in this study are included in the article. Further inquiries can be directed to the corresponding author.

## References

[1] Wang P., Lei H., Zhu X., Chen H., Fang D. (2019). Influence of manufacturing geometric defects on the mechanical properties of AlSi10Mg alloy fabricated by selective laser melting. J. Alloys Compd..

[2] Leary M., Mazur M., Williams H., Yang E., Alghamdi A., Lozanovvski B., Zhang X., Shidid D., Sternahl L.F., Witt G. (2018). Inconel 625 lattice structures manufactured by selective laser melting (SLM): Mechanical properties, deformation and failure modes. Mater. Des..

[3] Val C.G., Pallas A., Panadeiro V., Rodriguez A. (2020). A convolutional approach to quality monitoring for laser manufacturing. J. Intell. Manuf..

[4] Kwon O., Kim H.G., Ham M.J., Kim W., Kim G.H., Cho J.H., Kim N., Kim K. (2020). A deep neural network for classification of melt-pool images in metal additive manufacturing. J. Intell. Manuf..

[5] Guo N., Leu M.C. (2013). Additive manufacturing: Technology, applications and research needs. Front. Mech. Eng..

[6] Simchi A. (2006). Direct laser sintering of metal powders: Mechanism, kinetics and microstructural features. Mater. Sci. Eng. A.

[7] Qiu Y., Wu J., Chen A., Chen P., Yang Y., Liu R., Chen G., Chen S., Shi Y., Li C. (2020). Balling phenomenon and cracks in alumina ceramics prepared by direct selective laser melting assisted with pressure treatment. Ceram. Int..

[8] Yan A., Wang Z., Yang T., Wang Y., Ma Z. (2016). Microstructure, thermal physical property and surface morphology of W-Cu composite fabricated via selective laser melting. Mater. Des..

[9] Gu D., Shen Y. (2007). Balling phenomena during direct laser sintering of multi-component Cu-based metal powder. J. Alloys Compd..

[10] Herzog D., Seyda V., Wycisk E., Emmelmann C. (2016). Additive manufacturing of metals. Acta Mater..

[11] Yusuf S.M., Chen Y., Boardman R., Yang S., Gao N. (2017). Investigation on porosity and microhardness of 316L stainless steel fabricated by selective laser melting. Metals.

[12] Debroy T., Wei H.L., Zuback J.S., Mukherjee T., Elmer J.M., Milewski J.O., Beese A.M., Heid A.W., De A., Zhang W. (2018). Additive manufacturing of metallic components—process, structure and properties. Prog. Mater. Sci..

[13] Zhang H., Xu M., Kumar P., Li C., Liu Z., Zhang Y. (2021). Fatigue life prediction model and entropy generation of 304L stainless steel fabricated by selective laser melting. J. Mater. Process. Technol..

[14] Childs T.H.C., Hauser C., Badrossamay M. (2004). Mapping and modelling single scan track formation in direct metal selective laser melting. CIRP Ann..

[15] Yadroitsev I., Gusarov A., Yadroitsava I., Smurov I. (2010). Single track formation in selective laser melting of metal powders. J. Mater. Process. Technol..

[16] Dai D., Gu D. (2015). Tailoring surface quality through mass and momentum transfer modeling using a volume of fluid method in selective laser melting of tic/ alsi10mg powder. Int. J. Mach. Tools Manuf..

[17] Zöller C., Adams N.A., Adami S. (2023). Numerical investigation of balling defects in laser-based powder bed fusion of metals with Inconel 718. Addit. Manuf..

[18] Zhou X., Liu X., Zhang D., Shen Z., Liu W. (2015). Balling phenomena in selective laser melted tungsten. J. Mater. Process. Technol..

[19] Boutaous M., Liu X., Siginer D.A., Xin S. (2021). Balling phenomenon in metallic laser based 3D printing process. Int. J. Therm. Sci..

[20] Jia Q., Gu D. (2014). Selective laser melting additive manufacturing of Inconel 718 superalloy parts: Densification, microstructure and properties. J. Alloys Compd..

[21] Li R., Liu J., Shi Y., Wang L., Jiang W. (2012). Balling behavior of stainless steel and nickel powder during selective laser melting process. Int. J. Adv. Manuf. Technol..

[22] Gu D., Shen Y. (2009). Balling phenomena in direct laser sintering of stainless steel powder: Metallurgical mechanisms and control methods. Mater. Des..

[23] Yu G., Gu D., Dai D., Xia M., Ma C., Shi Q. (2016). On the role of processing parameters in thermal behavior, surface morphology and accuracy during laser 3D printing of aluminum alloy. J. Phys. D Appl. Phys..

[24] Costa A., Buffa G., Palmeri D., Pollara G., Fratini L. (2022). Hybrid prediction-optimization approaches for maximizing parts density in SLM of Ti6Al4V titanium alloy. J. Intell. Manuf..

[25] Cao L., Li J., Hu J., Liu H., Wu Y., Zhou Q. (2021). Optimization of surface roughness and dimensional accuracy in LPBF additive manufacturing. Opt. Laser Technol..

[26] Zhang M., Sun C., Zhang X., Goh P.C., Wei J., Hardacre D., Li H. (2019). High cycle fatigue life prediction of laser additive manufactured stainless steel: A machine learning approach. Int. J. Fatigue.

[27] Mehrpouya M., Gisario A., Rahimzadeh A., Nematollahi M., Baghbaderani K.S., Elahinia M. (2019). A prediction model for finding the optimal laser parameters in additive manufacturing of NiTi shape memory alloy. Int. J. Adv. Manuf. Technol..

[28] Vijayaraghavan V., Garg A., Lam J.S.L., Panda B., Mahapatra S.S. (2015). Process characterisation of 3D-printed FDM components using improved evolutionary computational approach. Int. J. Adv. Manuf. Technol..

[29] Shelhamer E., Long J., Darrell T. (2017). Fully convolutional networks for semantic segmentation. IEEE Trans. Pattern Anal. Mach. Intell..

[30] Zhao K., Liang X., Wang W., Yang P., Hao Y., Zhu Z. (2020). Multi-objective optimization of coaxial powder feeding laser cladding based on NSGA-II. Chin. J. Lasers.

[31] Dejene N.D., Lemu H.G., Gutema E.M. (2024). Effects of process parameters on the surface characteristics of laser powder bed fusion printed parts: Machine learning predictions with random forest and support vector regression. Int. J. Adv. Manuf. Technol..

[32] Cao C., Zhao Y., Song Z., Dai D., Liu Q., Zhang X., Meng J., Gao Y., Zhang H., Liu G. (2022). Prediction and optimization of surface roughness for laser-assisted machining SiC ceramics based on improved support vector regression. Micromachines.

[33] Wang J., Xu J., Lu Y., Xie T., Peng J., Chen J., Xu Y. (2023). Prediction of deposition layer morphology dimensions based on PSO-SVR for laser–arc hybrid additive manufacturing. Coatings.

[34] Liang R., Yu R., Luo Y., Zhang Y. (2019). Machine learning of weld joint penetration from weld pool surface using support vector regression. J. Manuf. Process..

[35] Xing Y., Li F. (2020). Research on the influence of hidden layers on the prediction accuracy of GA-BP neural network. J. Phys. Conf. Ser..

[36] Xia C., Pan Z., Polden J., Li H., Xu Y., Chen S. (2022). Modelling and prediction of surface roughness in wire arc additive manufacturing using machine learning. J. Intell. Manuf..

[37] Katagiri J., Kusano M., Minamoto S., Kitano H., Daimaru K., Tsujii M., Watanabe M. (2023). Melt pool shape evaluation by single-track experiments and finite-element thermal analysis: Balling and lack of fusion criteria for generating process window of Inconel738LC. Materials.

[38] Zhu J., Borisov E., Liang X., Farber E., Hermans M.J.M., Popovich V.A. (2021). Predictive analytical modelling and experimental validation of processing maps in additive manufacturing of nitinol alloys. Addit. Manuf..

[39] Yan Z., Liu S., Sun Z., Li K., Su N., Yang G. (2024). In situ X-ray imaging and quantitative analysis of balling during laser powder bed fusion of 316L at high layer thickness. Mater. Des..

